# Supplementary material for: Electrophysiological and Imaging Biomarkers to Evaluate Exercise Training in Patients with Neuromuscular Disease: A Systematic Review
Source: J Clin Med. 2023 Oct 29;12(21):6834. doi: 10.3390/jcm12216834 (PMC10647337; doi:10.3390/jcm12216834)
Supplement: Supplementary file 1 [file jcm-12-06834-s001.zip › jcm-2649528-supplementary.pdf]

Supplementary Materials

A. Search strings

Table S1. Search string in PubMed

Database	PubMed	09-01-2023
#1 Participants	("neuromuscular diseases"[MeSH Terms] OR "neuromuscular disease"[Title/Abstract] OR 'neuromuscular disorder'[Title/Abstract] OR 'polymyositis'[Title/Abstract] OR 'dermatomyositis'[Title/Abstract] OR 'mitochondrial myopathies'[Title/Abstract])	345,286
#2 Intervention	("Exercise Therapy"[MeSH Terms] OR "exercise"[MeSH Terms] OR "exerci*"[Title/Abstract] OR "exercise training"[Title/Abstract] OR "Exercise Therapy"[Title/Abstract])	492,777
#3 Outcome	("magnetic resonance spectroscopy"[MeSH Terms] OR "ultrasonography"[MeSH Terms] OR "magnetic resonance imaging"[MeSH Terms] OR "electromyography"[MeSH Terms] OR "spectroscopy, near infrared"[MeSH Terms] OR "MRI"[Title/Abstract] OR "MRS"[Title/Abstract] OR "EMG"[Title/Abstract] OR 'magnetic resonance spectroscopy'[Title/Abstract] OR 'magnetic resonance imaging'[Title/Abstract] OR 'electromyography'[Title/Abstract] OR 'ultrasound'[Title/Abstract] OR 'near infrared spectroscopy'[Title/Abstract])	1,608,184
	#1 AND #2 AND #3	1,359
	AND (humans[Filter])	1,294
	NOT 'review'	1,102
	NOT 'Carpal Tunnel Syndrome'[Title/Abstract]	1,065
	NOT 'Case report'	**751**

Table S2. Search string in EMBASE

Database	EMBASE	09-01-2023
#1 Participants	'neuromuscular diseases'/exp OR 'neuromuscular disease':ab,ti OR 'neuromuscular disorder':ab,ti OR 'polymyositis':ab,ti OR 'dermatomyositis':ab,ti OR 'mitochondrial myopathies':ab,ti	251740
#2 Intervention	'kinesiotherapy'/exp OR 'exercise'/exp OR 'exerci*':ab,ti OR 'exercise training':ab,ti OR 'exercise therapy':ab,ti	646200
#3 Outcome	'nuclear magnetic resonance spectroscopy'/exp OR 'echography'/exp OR 'nuclear magnetic resonance imaging'/exp OR 'electromyography'/exp OR 'near infrared spectroscopy'/exp OR 'mri':ab,ti OR 'mrs':ab,ti OR 'emg':ab,ti OR 'magnetic resonance spectroscopy':ab,ti OR 'magnetic resonance imaging':ab,ti OR 'electromyography':ab,ti OR 'ultrasound':ab,ti OR 'near-infrared spectroscopy':ab,ti	2,574,171
	#1 AND #2 AND #3	1,945
	NOT ('animal'/exp NOT 'human'/exp)	1857
	NOT 'review'	1412
	NOT 'case report'	**812**

Table S3. Search string in CINAHL

Database	CINAHL	09-01-2023
#1 Participants	MH neuromuscular disease OR TI neuromuscular disease OR AB neuromuscular disease OR TI neuromuscular disorder OR AB neuromuscular disorder OR TI polymyositis OR AB polymyositis OR TI dermatomyositis OR AB dermatomyositis OR TI mitochondrial myopathies OR AB mitochondrial myopathies	4,629
#2 Intervention	MH exercise training OR MH exercise OR TI exercise OR AB exercise OR TI exercise training OR AB exercise training OR TI Exercise Therapy OR AB Exercise Therapy	165,794
#3 Outcome	MH magnetic resonance imaging OR MH magnetic resonance spectroscopy OR MH electromyography OR MH echography OR MH near infrared spectroscopy OR TI MRI OR AB MRI OR TI MRS OR AB MRS OR TI EMG OR AB EMG OR OR TI magnetic resonance imaging OR AB magnetic resonance imaging OR TI magnetic resonance spectroscopy OR AB magnetic resonance spectroscopy OR TI electromyography OR AB electromyography OR TI ultrasound OR AB ultrasound OR TI near-infrared spectroscopy OR AB near-infrared spectroscopy	278,973
	#1 AND #2 AND #3	**34**

Table S4. Search string in Cochrane

**Database**	**Cochrane**	**09-01-2023**
**#1 Participants**	"neuromuscular disease" OR 'neuromuscular disorder' OR 'polymyositis' OR 'dermatomyositis' OR 'mitochondrial myopathies' in Title Abstract Keyword	136
**#2 Intervention**	"exerci*" OR "exercise training" OR "Exercise Therapy" in Title Abstract Keyword	208
**#3 Outcome**	"MRI" OR "MRS" OR "EMG" OR 'magnetic resonance spectroscopy' OR 'magnetic resonance imaging' OR 'electromyography' OR 'ultrasound' OR 'near infrared spectroscopy' in Title Abstract Keyword	355
	#1 AND #2 AND #3	**22**

B. Quality Assessments

Table S5. NIH Pre-Post Quality Assessment

**First author et al. (Year)**	**1**	**2**	**3**	**4**	**5**	**6**	**7**	**8**	**9**	**10**	**11**	**12**	**Total Score**	**Quality Rating**
Alexanderson (1999)	Y	N	NR	NR	NR	N	N	NR	Y	Y	N	NA	25%	**Poor**
Alexanderson (2000)	Y	Y	N	Y	NR	N	N	NR	Y	Y	N	NA	42%	**Poor**
El Mhandi (2007)	Y	Y	Y	Y	NR	Y	Y	NR	Y	Y	N	NA	67%	**Fair**
Lott (2021)	Y	Y	Y	N	NR	Y	Y	NR	Y	Y	N	NA	58%	**Fair**
Porcelli (2015)	Y	Y	Y	NR	NR	Y	Y	NR	Y	Y	N	NA	58%	**Fair**
Spector (1996)	Y	Y	Y	Y	NR	Y	N	NR	Y	Y	N	NA	58%	**Fair**
Taivassalo (1998)	Y	N	Y	NR	NR	Y	Y	NR	Y	Y	N	NA	50%	**Fair**
Taivassalo (2001)	Y	N	Y	NR	NR	Y	N	NR	Y	Y	Y	NA	50%	**Fair**
Töllback (1999)	Y	Y	Y	Y	NR	Y	Y	N	N	Y	N	NA	58%	**Fair**
Trenell (2005)	Y	N	Y	NR	NR	Y	Y	N	Y	Y	N	NA	50%	**Fair**
Westerberg (2018)	Y	Y	Y	Y	NR	Y	Y	N	Y	Y	N	NA	67%	**Fair**

Table S6. Cochrane ROB-2 Quality Assessment

**First author et al. Year**	**1.1**	**1.2**	**1.3**	**Risk**	**2.1**	**2.2**	**2.3**	**2.4**	**2.5**	**2.6**	**Risk**
Bulut (2022)	Y	Y	Y	Some concerns	Y	N	PY	N	Y	N	High
Burns (2017)	Y	Y	PN	Low	N	Y	Y	N	PN	-	Low
Chung (2007)	Y	Y	PN	Low	N	N	-	N	Y	Y	Some concerns
Janssen (2016)	Y	Y	PN	Low	Y	N	PY	N	Y	N	High
Rahbek (2016)	Y	Y	N	Low	PY	N	PY	N	Y	N	High

**First author et al.**	**3.1**	**3.2**	**3.3**	**3.4**	**Risk**	**4.1**	**4.2**	**4.3**	**4.4**	**4.5**	**Risk**	**5.1**	**5.2**	**5.3**	**Risk**	**Overall Risk**
Bulut	N	N	Y	Y	High	N	N	N	-	-	Low	Y	PN	PN	Low	**High**
Burns	N	PY	-	-	Low	PN	N	N	-	-	Low	Y	PN	PN	Low	**Low**
Chung	N	PN	PN	-	Low	N	N	N	-	-	Low	Y	PN	PN	Low	**Some concerns**
Janssen	N	PN	PN	-	Low	N	PN	N	-	-	Low	PY	PN	PN	Low	**High**
Rahbek	N	PN	PN	-	Low	N	PN	N	-	-	Low	PY	PN	PN	Low	**High**

Table S7. Cochrane ROBINS-1 Quality Assessment

**First author et al. Year**	**1.1**	**1.2**	**1.3**	**1.4**	**1.5**	**1.6**	**1.7**	**1.8**	**Risk**	**2.1**	**2.2**	**2.3**	**2.4**	**2.5**	**Risk**
Taivassalo (1999)	Y	N	-	PN	-	N	-	-	Serious	N	-	-	Y	-	Low

**First author et al.**	**3.1**	**3.2**	**3.3**	**Risk**	**4.3**	**4.4**	**4.5**	**4.6**	**Risk**	**5.1**	**5.2**	**5.3**	**5.4**	**5.5**	**Risk**
Taivassalo	Y	Y	N	Low	NI	Y	Y	-	Low	PY	N	N			Low

**First author et al.**	**6.1**	**6.2**	**6.3**	**6.4**	**Risk**	**7.1**	**7.2**	**7.3**	**Risk**	**Overall Risk**
Taivassalo	Y	Y	Y	NI	Serious	N	Y	PN	Serious	**Serious**

C. Functional measure outcomes

Table S8. Baseline and follow-up measurement functional tests

Study	NMD	Endurance / Resistance functional test	Functional Test	Baseline	After intervention
**Mhandi [40]**	CMT	Endurance + Resistance	Functional time-scored activity e.g., descending stair	-5.5 ± 0.9 s	**Significant improvement except for 1/6 activity ($p < 0.002 – 0.007$)**
		Resistance	Isokinetic muscle strength (Nm) knee extension/flexion		**$p = 0.03$ and $p = 0.003$.**
		Endurance	Cardiorespiratory cycle test ($VO_{2\ peak}$, P_{max}, HR_{max}, $Lactate_{max}$)		**$p = 0.02$, 0.003,** 0.24, 0.11
**Burns [32]**	CMT	Endurance	CMTPedS Score<br>(0 - 44)	13 ± 7 / 13 ± 7 (resistive/sham group resp.)	13 ± 7 / 12 ± 9 (resistive/sham group resp.) $p = 0.84$
		Resistance	Strength dorsiflexion		**$p = 0.041$**
		Endurance + Resistance	Gait (foot drop, ankle power at push off, knee flexion in swing, hip flexion in swing)		$p = 0.81$, 0.70, 0.98, 0.18
**Alexanderson [34]**	PM and DM	Endurance	Functional index in myositis (0 – 64)	Right side – 48.5 (32-64)<br>Left side – 47.5 (28-63)	**Right side – 57 (41-64) ($p < 0.05$)**<br>**Left side – 57 (42-63) ($p < 0.05$)**
		Endurance	Walking distance	312 (81-422)	**404 (124-549) ($p < 0.05$)**
**Alexanderson [35]**	PM and DM		Functional index in myositis (0 – 64)	right side – 52 (32-62)<br>left side – 50 (33-62)	**Functional index significantly improved ($p < 0.05$ right, $p < 0.01$ left)**
**Chung [31]**	PM and DM (only control)	Endurance	Functional index in myositis	46.3 (36.0-53.4)	**51.8 (38.4-57.3) ($p = 0.015$)**
		Resistance	AFPT scores	30 (24-414) s	Not significant
		Resistance	Strength individual muscles		Not significant
**Taivassalo [44]**	MM	Endurance	Aerobic capacity	4.39 ± 1.55 METs	**5.79 ± 1.72 METs ($p < 0.01$)**
		Endurance	Exercise tolerance	12.3 ± 6 min	**16.2 ± 7.0 min ($p < 0.02$)**
**Taivassalo [33]**	MM and NMM	Endurance	Aerobic capacity (METs)	MM: 4.6 ± 1.6<br>NMM: 4.9 ± 1.4	**MM: 5.9 ± 1.9 ($p < 0.01$)**<br>**NMM: 5.67 ± 1.7 ($p < 0.01$)**

**Taivassalo [38]**	MM	Endurance	Cardiorespiratory cycle test (Work capacity, VO_2, Cardiac output, a-vO2, ΔQ/ΔVO2)	45 ± 21 watts	**59 ± 27 watts (p < 0.05)**<br>**Work capacity, VO_2 and a-vO2 significant,** Cardiac output and ΔQ/ΔVO2 non-significant
**Trenell [39]**	MM	Endurance<br>Endurance	6MWT<br>Progressive exercise test (MHRR VO_2, oxygen uptake efficiency slope, Peak Watts)	508 ± 142 m	**555 ± 118 m (p < 0.05)**<br>**Oxygen uptake efficiency slope and peak watts significant (p < 0.05)**, MHRR VO2 non-significant.
**Porcelli [41]**	MM and McA	Endurance + Resistance	Incremental exercise test (Peak work rate (W))	MM: 72 ± 13<br>McA: 73 ± 13	**MM: 88 ± 15 (p < 0.05)**<br>**McA: 89 ± 12 (p < 0.05)**
		Endurance	Low-intensity constant work rate exercise test (work rate (%W_{peak}))	MM: 54 ± 5<br>McA: 59 ± 5	**MM: 45 ± 6 (p < 0.05)**<br>**McA: 48 ± 4 (p < 0.05)**
**Rahbek [29]**	MG	Endurance + Resistance	6MWT, STS, B&B, and SCT	PRT: 527 ± 100 m<br>ET: 617 ± 96 m	PRT: 6MWT (562 ± 82 m, p = 0.08), **STS (p = 0.04), B&$B_{dominant}$ (p = 0.01),** SCT (p = 0.08)<br>ET: 6MWT (624 ± 95, p = 0.60), **STS (p = 0.04),** B&$B_{dominant}$ (p = 0.56), SCT (p = 0.23)
		Resistance	Isokinetic strength		**PRT: knee extensor (p = 0.02) and shoulder abductor (p = 0.05) significant**<br>AT: non-significant
		Endurance + Resistance	QMG	PRT: 5.5 (2-14)<br>ET: 6.5 (0-17)	PRT: 4.5 (4 - 8), p = 0.50<br>ET: 4.5 (0 - 22), p = 0.65
**Westerberg [42]**	MG	Endurance + Resistance	TUG, 12MWT, 30SCST, Jamar	n.a.	**30SCST median change +2 (p = 0.0039),** TUG, 12MWT and Jamar non-significant change (p > 0.05).
		Endurance + Resistance	QMG & MGC		Median QMG change from 3 to 1 (p > 0.05) and **median MGC change from 3 to 2 (p = 0.043)**
		Resistance	Isometric muscle force	Quadriceps: 25.2 ± 4.4 kg<br>Biceps brachii: 21.0 ± 6.0 kg	**Quadriceps: 30.2 ± 3.8 kg (p = 0.014)**<br>Biceps brachii: 21.9 ± 5.6 kg (p = 0.58)
**Janssen [17, 23][1]**	FSHD1	Endurance	6MWT	ET: 388 (136-630) m/ UC: 436 (80-708) m	ET: 420 (159 - 605) m / UC: 430 (90 - 800)
**Lott [43]**	DMD	Endurance + Resistance	4-stair ascent / descent:	3.7 s up and 3.4 s down	+ 13.5% up (p = 0.09) and + **22.7% down (p < 0.05)**
		Resistance	Peak isometric strength (peak torque)	45 Nm knee extensors and 49 Nm knee flexors	**+ 20.6% for knee extensors (p < 0.01) and 14.3% for knee flexors (p < 0.05)**
**Bulut [30]**	DMD	Endurance	6MWT	395.3 ± 46.6 m / control: 421.7 ± 64.4 m	**413.0 ± 52.3 m / control: 393.8 ± 68.2 m (p < 0.001)**
		Endurance + Resistance	Motor function measure	83.2 ± 6.1 / control: 82.3 ± 10.2	**86.9 ± 4.0 / control: 80.4 ± 9.4 (p = 0.006)**
**Spector [36]**	PPMA	Resistance	3RM test	n.a.	**+ 41 ± 16 % for leg press, + 61 ± 50% for knee extension exercises, + 54 ± 8 % for arm press and +71 ± 18 % for arm extension exercises (p < 0.05)**

**Tollbäck [37]**	MD	Resistance	1RM test	16.4 ± 3.4 kg	**21.8 ± 2.6 kg (p = 0.0002)**

Bold is a significant difference between baseline and end intervention.

[1] Results of the functional test have only been reported in another study [17].

[2] between group (intervention vs control) comparison p-value ($p < 0.05$ is significant).

Abbreviations: NMD = neuromuscular disease; CMT = Charcot-Marie-Tooth disease; PM = Polymyositis; DM = dermatomyositis; MM = Mitochondrial myopathy; NMM = chronic nonmetabolic myopathies; McA = McArdle disease; MG = Myasthenia Gravis; FSHD1 = Facioscapulohumeral muscular dystrophy type 1; DMD = Duchenne Muscular Dystrophy; PPMA = postpolio muscular atrophy; MD = myotonic dystrophy; $VO_{2\,peak}$ = maximal oxygen consumption; P_{max} = maximum power; HR_{max} = maximum heart rate; CMTPedS = Charcot-Marie-Tooth disease Pediatric Scale; AFPT = aggregate functional performance time; METs = metabolic equivalents; a-vO2 = arteriovenous oxygen uptake; Q = cardiac output; 6MWT = 6-minute walk test; STS = 30s sit to stand test; B&B = box and block test; SCT = stair climb test; MHRR = maximum heart rate reserve; TUG = Timed Up and Go; 12MWT = 12-minute walk test ; 30SCST = 30-second Chair Stand Test; Jamar = handgrip strength test; QMG = Quantitative Myasthenia Gravis score; MGC = Myasthenia Gravis composite; 3RM = three repetition maximum; 1RM = one repetition maximum; PRT = progressive resistance training; ET = endurance training; UC = usual care.
